# A Dynamic Real Time In Vivo and Static Ex Vivo Analysis of Granulomonocytic Cell Migration in the Collagen-Induced Arthritis Model

**DOI:** 10.1371/journal.pone.0035194

**Published:** 2012-04-18

**Authors:** Ruth Byrne, Eva Rath, Anastasiya Hladik, Birgit Niederreiter, Michael Bonelli, Sophie Frantal, Josef S. Smolen, Clemens Scheinecker

**Affiliations:** 1 Divison of Rheumatology, Internal Medicine III, Medical University of Vienna, Waehringer Guertel, Vienna, Austria; 2 Center for Medical Statistics, Informatics and Intelligent Systems, Medical University of Vienna, Waehringer Guertel, Vienna, Austria; University of São Paulo, Brazil

## Abstract

Neutrophilic granulocytes and monocytes (granulomonocytic cells; GMC) drive the inflammatory process at the earliest stages of rheumatoid arthritis (RA). The migratory behavior and functional properties of GMC within the synovial tissue are, however, only incompletely characterized. Here we have analyzed GMC in the murine collagen-induced arthritis (CIA) model of RA using multi-photon real time in vivo microscopy together with ex vivo analysis of GMC in tissue sections.

GMC were abundant as soon as clinical arthritis was apparent. GMC were motile and migrated randomly through the synovial tissue. In addition, we observed the frequent formation of cell clusters consisting of both neutrophilic granulocytes and monocytes that actively contributed to the inflammatory process of arthritis. Treatment of animals with a single dose of prednisolone reduced the mean velocity of cell migration and diminished the overall immigration of GMC.

In summary, our study shows that the combined application of real time in vivo microscopy together with elaborate static post-mortem analysis of GMC enables the description of dynamic migratory characteristics of GMC together with their precise location in a complex anatomical environment. Moreover, this approach is sensitive enough to detect subtle therapeutic effects within a very short period of time.

## Introduction

Rheumatoid arthritis (RA) is a systemic inflammatory disease characterized by chronic articular inflammation and progressive destruction of the joints. Affected joints display an accumulation of a variety of different cell types [1]. While in human RA monocytes and lymphocytes are the prevalent hematopoietic cells in the synovial membrane, activated granulocytes are highly present in the synovial fluid [2] and, by virtue of their mediator release and enzymatic machinery, can exert significant inflammatory and destructive effects in arthritis [3]. Moreover, experimental models of RA are characterized by the presence of granulocytes in the synovial membrane [4], [5].

Thus, neutrophils and monocytoid cells (granulomonocytic cells or GMC) are important participants in joint inflammation; indeed, they constitute the majority of immigrating cells particularly at early stages of the disease [5]. The synovial tissue in inflamed joints is infiltrated with a broad variety of immune cells with different functional roles in the pathogenesis of the disease. Their presumed cognate or non-cognate interactions may result in the formation of cell clusters and even lymphoid follicle-like structures. Acquisition of more detailed knowledge of the role of GMC and other cells in RA is, thus, of substantial importance for understanding mechanisms that contribute to the disease state and will contribute to the identification of new therapeutic targets, the development of new drugs and the assessment of their therapeutic potential in RA. However, key features of the behavior and properties of GMC, such as the dynamics of cell immigration and migration within inflamed joint tissues and spatiotemporal aspects of the functional activities of these cells in such tissue sites, have not been well described to date [6], [7], leaving important gaps in our understanding of the inflammatory process.

Recently, advanced light microscopy tools have revolutionized our ability to analyze more directly the migratory behavior of immune cells in the natural tissue environment. Confocal and multi-photon microscopy instruments, in particular, have been widely used to study the biology of single-cell dynamics in tissue explants and living mice. Although initially established for the analysis of immune cell interaction in secondary lymphoid organs, in vivo imaging has been increasingly adopted for dynamic cell analysis in peripheral tissues including organs such as the liver, kidney, and lung, as well as various epithelial surfaces [8].

The collagen-induced arthritis model (CIA) currently represents the most extensively used small animal model of RA. CIA is induced in susceptible mouse strains by immunization with heterologous type II collagen (CII) in adjuvant, leading to a cross-reactive immune response to murine type II collagen. Both T cell and B cell responses to CII are required to establish the disease, and there is a significant role for auto-reactive antibodies that form immune complexes and are capable of inducing inflammatory pathways via the activation of the complement cascade [9]. GMC are among the first cells that can be detected in the synovial tissue at the earliest stages of CIA [6].

Here we have employed this disease model in a combined approach of multi-photon real time in vivo microscopy and detailed post-mortem histomorphological analyses, to gain a better understanding of the innate immune cell dynamics in the early stages of joint disease. In addition, we evaluated the potential of this approach to explore the immediate effects of a therapeutic compound, prednisolone, on the migratory behavior of cells, seeking a link between therapeutic efficacy and alterations in spatiotemporal behavior of inflammatory cells in the joint.

## Results

### Early detection of GMC in the synovial tissue of animals with CIA

**Figure 1:**
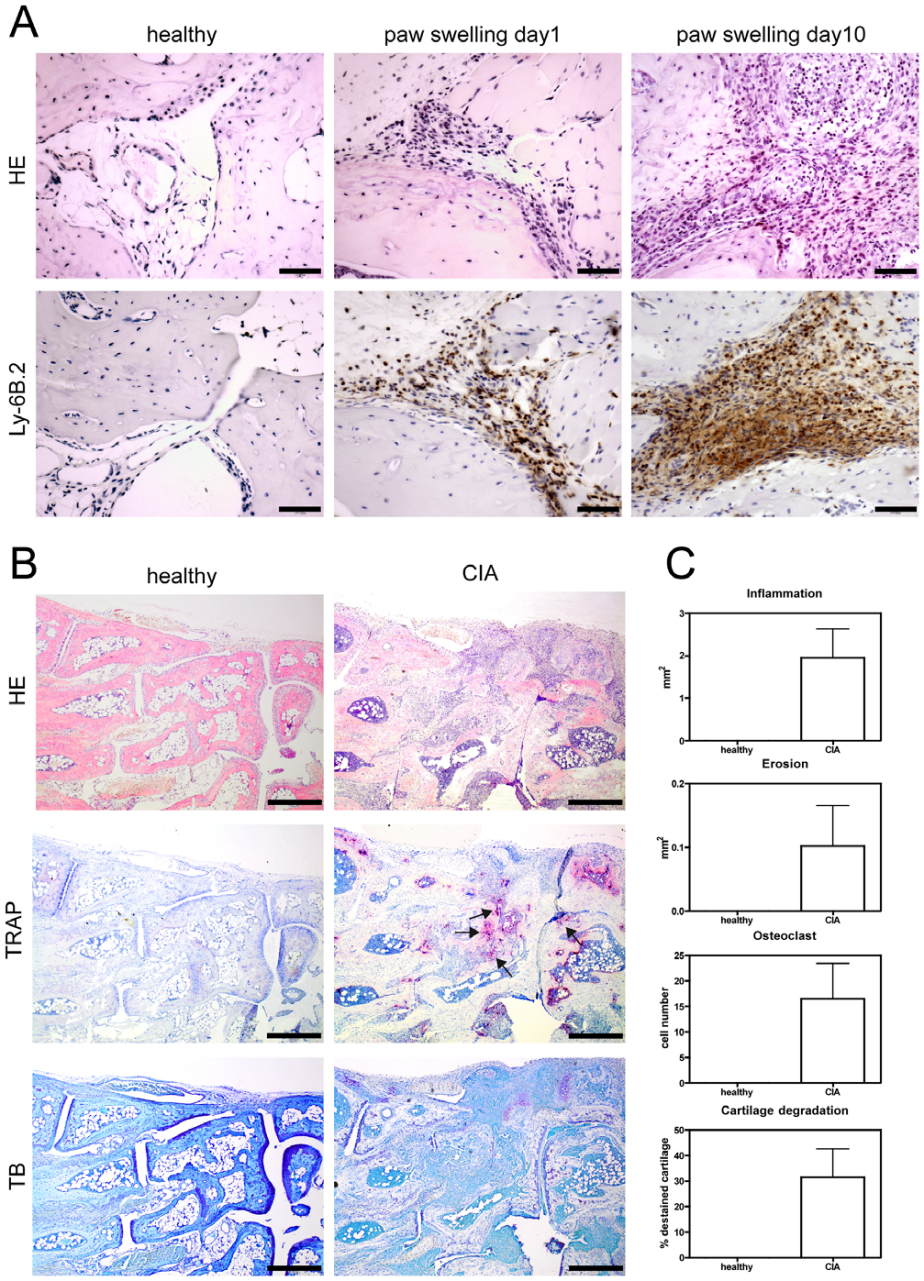
Analysis of GMC migration and histomorphological assessment of arthritis in animals with CIA. A) Paraffin sections were stained with HE and anti-Ly-6B.2 mAb. In contrast to healthy animals GMC cells can be detected in animals with CIA at the earliest time-points (paw swelling day 1) of clinical signs of arthritis and are abundant at later (paw swelling day 10) time-points. Scale bars represent 50 µm. Representative pictures are shown from experiments that were performed in three healthy animals and three animals with CIA. B) Representative samples of paraffin sections from hind paws of three healthy control animals and three animals with CIA stained for HE, TRAP and TB are shown. Arrows point to areas of bone erosions. Scale bars represent 500 µm. C) Histomorphological signs of arthritis were quantified in three CIA subjects (day 11±6 of paw swelling) as compared to three healthy control subjects. Areas of inflammation, bone erosions, osteoclasts and cartilage degradation can be detected in CIA subjects but not in healthy control animals. Graph bars represent mean values ± s.e.m.

In order to determine the earliest time point of detectable GMC migration into the synovial tissue during CIA, we sacrificed animals before, immediately after the onset of paw swelling (day 1) and after a period of sustained paw swelling (day 10). We prepared paraffin sections of hind paws and stained them with hematoxylin-eosin (HE) and the GMC marker Ly-6B.2. We found that Ly-6B.2 positive GMC were hardly detectable in healthy animals (Figure 1A). However, we detected GMC within the synovial tissue of animals with CIA after 1 day of paw swelling. Thereafter, numbers of GMC substantially increased over time and represented the majority of inflammatory cells within the synovial tissue at later time points.

### Histomorphological analysis

We evaluated histomorphological signs of arthritis from paraffin sections of affected hind paws from 3 animals after 11±6 days of paw swelling using a digital image analysis system. We detected inflammation in tissues from animals with CIA, but not in those from healthy control animals. Such inflammation affected the entire tarsal area and covered on average 1.9±0.6 mm^2^ or 13.5±9.4% of the entire section (Figure 1B, 1C).

We further determined the area of bone erosions and quantified the number of synovial osteoclasts adjacent to bone erosions. In contrast to healthy control subjects without bone erosions, we found an increasing area of bone erosion during prolonged periods of paw swelling in animals with CIA (mean area of erosions in the bone area: 0.10±0.06 mm^2^ or 3±3.3% of the bone area in the section). In addition, we observed osteoclasts at the articular bone surface of CIA animals (16.5±13.7/section), whereas none were seen in the joints of healthy control animals (Figure 1B, 1C). We quantified cartilage damage as reduced proteoglycan content indicated by decreased toluidine blue (TB) staining intensity as described previously [10]. On average, we found that the destained cartilage area in CIA subjects represented 31.5±22.3% of the whole cartilage area as compared to no destaining in samples from healthy animals (Figure 1B, 1C).

### GMC actively migrate into the synovial tissue of LysM-EGFP C57BL/6 animals with CIA

We analyzed the migratory behavior of GMC by real time in vivo imaging experiments that were performed in LysM-EGFP transgenic mice. These animals express the EGFP fluorescent protein under the lysozyme promoter. Therefore, all GMC can be detected in the green fluorescence channel [11] and easily analyzed by fluorescence microscopy or flow cytometry. We analyzed LysM-EGFP C57BL/6 animals with CIA at early time points of arthritis (day 7±3 of paw swelling) and compared them to healthy control subjects.

Clearly, physiology is disturbed to a certain extent by any intravital technique that includes surgery. Inevitably this intrusion is accompanied by reactive inflammation and some degree of fluid leakage. We, therefore, paid attention to only dissect the skin covering the area of one to two metatarsal joints but not the periarticular and/or intraarticular synovial tissue and did so without opening the joint capsule. In addition, we kept the area of joint preparation as small as possible and carefully sealed the edges of the incision with high-temperature cautery.

**Figure 2:**
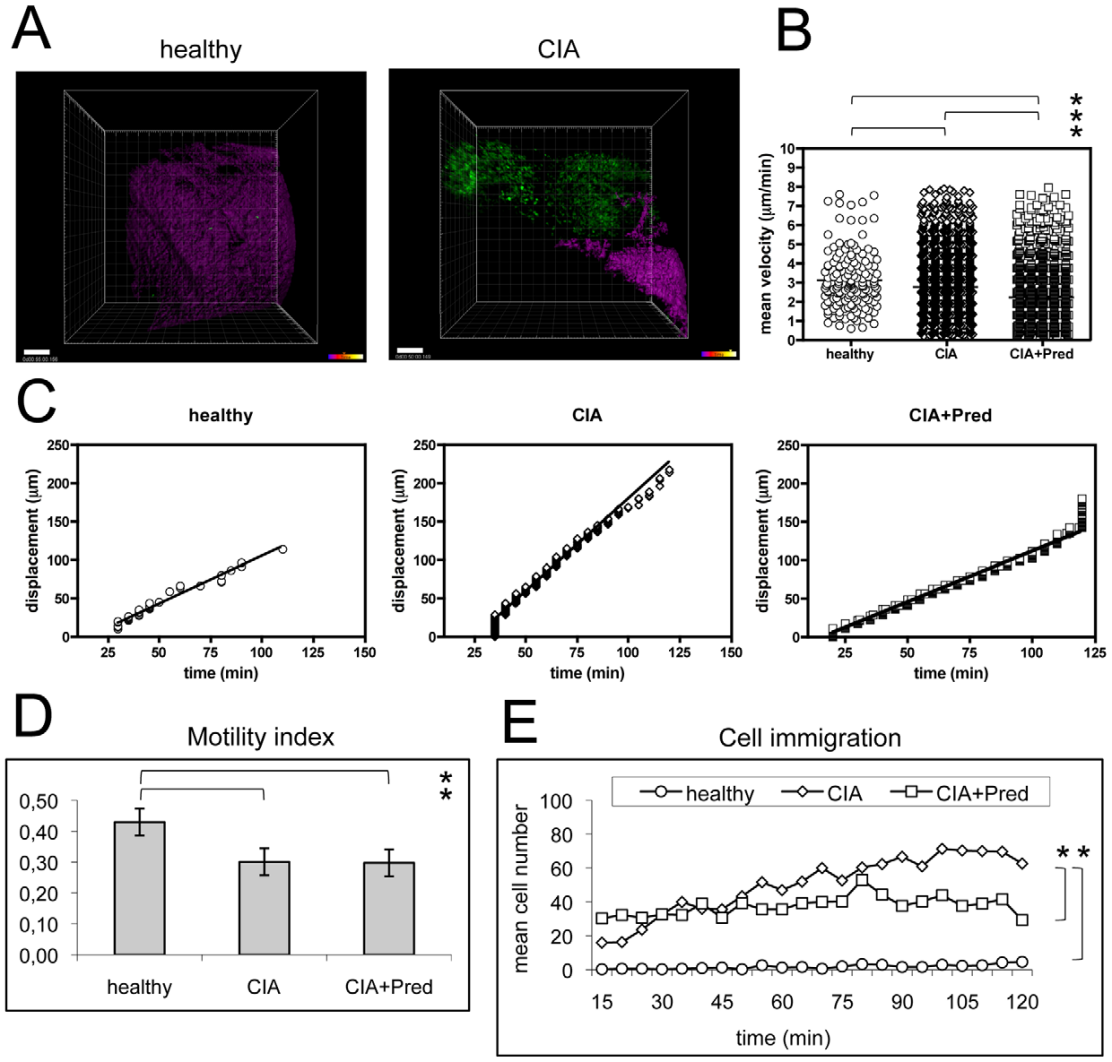
Kinetics of cell migration. A) Comparative real time in vivo analysis of cell migration in healthy versus CIA subjects were performed in C57BL/6 lysM-EGFP animals. GMC cells with the expression of EGFP (green) are rarely found in healthy tissue, whereas they are abundant in the synovial tissue of CIA subjects. The magenta signal displays a second harmonic generation (SHG) of collagen structures (see also Movie S1). Scale bars (left corner) represent 100 µm. Representative pictures are shown from movies that were made in three healthy control subjects and three subjects with CIA. B)–D): Quantification of cell velocity (b), displacement over time (c) and the motility index (total displacement/cumulative path length) (d) are shown for GMC cells in healthy subjects, in CIA subjects (CIA) and in CIA subjects treated with prednisolone (CIA+Pred). For (b) and (c), data points represent individual cells (healthy: n = 142; CIA: n = 4055; CIA+Pred: n = 3787) compiled from three independent imaging sessions involving individual joints of three animals. Mean values±s.e.m. are shown. Graph bars show mean±s.e.m. (d). Asterisks (*) indicate significant p values (<0.05). E) Cell immigration describes the number of cells immigrating into the ROI at each time point. Few cells immigrate in healthy subjects. CIA diseased subjects start with high numbers of GMC cells and increasing numbers of cells immigrate into the scanned area. Prednisolone stabilizes cell immigration to comparable numbers of cells immigrating at each time point. Data are mean values from experiment that were performed in three healthy controls, three CIA subjects and three CIA+Prednisolone subjects. Asterisks (*) indicate significant p values (<0.05).

Afterwards, we analyzed the inflamed tissue area by 2-photon laser microscopy. We identified EGFP^+^ GMC within the synovial tissue and performed cell imaging over a time period of 2 hours in a predefined region of interest (ROI). We rarely detected EGFP^+^ GMC in the synovial tissue of healthy control subjects suggesting that surgery by itself did not induce the migration of GMC into the ROI. In contrast, we observed abundant EGFP^+^ GMC in the synovial tissue of inflamed joints (Figure 2A, Movie S1, and Movie S2).

To describe cell migration behavior we determined velocity, displacement over time, motility index (MI), and cell abundance over time in three healthy subjects, three CIA subjects and three CIA subjects who received a single i.v. injection of 0.25 mg prednisolone.

#### Cell velocity

We found that the average cell migration speed of GMC through a predefined ROI was significantly decreased in CIA animals (2.75±1.17 µm/min) as compared to the rare cells we could visualize in the joints of healthy controls (3.11±1.51 µm/min; p<0.001) (Figure 2B, Movie S1, and Movie S2), suggesting that in healthy animals cells were trafficking accidentally through the joint, while in arthritic mice cells were more engaged within the joint tissue. Along these lines, we found that the average track duration was significantly increased in CIA subjects (49±15 min) as compared to healthy controls (42±15 min; p<0.0001). We excluded cell flow in blood vessels from these analyses since the chosen time intervals of z-stack scanning were too long to allow for the detection of fast moving cells in the blood stream.

Although we avoided collateral tissue damage as much as possible we observed a small but significant (p<0.0001) increase in the average cell speed in all experiments during the 2 h period of imaging suggesting that potential minor tissue damage due to the surgical procedure and/or laser emission to a certain extent affects GMC migration; however, this effect was similar across all types of experiments, whether in healthy or CIA animals.

Together the data indicate that in arthritic animals GMC populate the joint in highly increased numbers and show a reduced cell speed through the inflamed tissue, staying within this tissue for a prolonged time period when compared with healthy animals.

#### Random versus directed cell movement

We compared the migratory dynamics of GMC in CIA with healthy subjects and observed an increased spatial displacement over time (Figure 2C). We calculated the motility index (MI) by dividing the distance a cell traveled from its starting point by the track length. Values of >0.8 are commonly associated with a directed cell movement - as for example due to chemotaxis - whereas values of <0.5 are consistent with random cell migration [12]. We found the MI to be on average <0.5 for both healthy subjects and animals with CIA, but nevertheless still significantly higher for GMC in healthy subjects (0.43±0.19) as compared to GMC in CIA subjects (0.3±0.16; p = 0.009) (Figure 2D). Moreover, we found the relative proportion of cells with a MI>0.8 to be higher in healthy subjects (6.1%) than in CIA subjects (0.8%). Altogether, the migratory dynamics indicate a random migration of GMC in CIA as compared to healthy subjects.

#### Cell immigration and abundance

In healthy animals we observed very few cells that immigrated into the scanned area throughout the 2 h scanning period (Figure 2E). In contrast, we found that the number of immigrating cells in CIA animals was much higher from the beginning of the 2 h scanning period onward, and that GMC continued to accumulate in the scanned area. Thereby absolute cell immigration over time was found to be significantly lower in healthy subjects as compared to CIA subjects (Table 1a). These data indicate a continuous accumulation and confinement of GMC over time in the synovial tissue, where they travel at low cell speed and with a random migration pattern.

**Table 1:** Immigration of GMC over time.

a. Comparison of values at starting time point.			
	value	s.e.m.	p-value
healthy	−16.05	15.78	0.34
CIA	15.69	11.16	0.16
CIA+Pred	16.19	15.78	0.34

b. Comparison of slope curves (factor time).			
	Value	s.e.m.	p-value
healthy	−2.38	0.34	<0.0001
CIA	2.54	0.24	<0.0001
CIA+Pred	−2.12	0.34	<0.0001

Mean values±s.e.m. were calculated using a linear mixed-effects model including the random factor animal and the interaction between group and time.

### GMC aggregates in the synovial tissue

#### Analysis of GMC cluster formation

**Figure 3:**
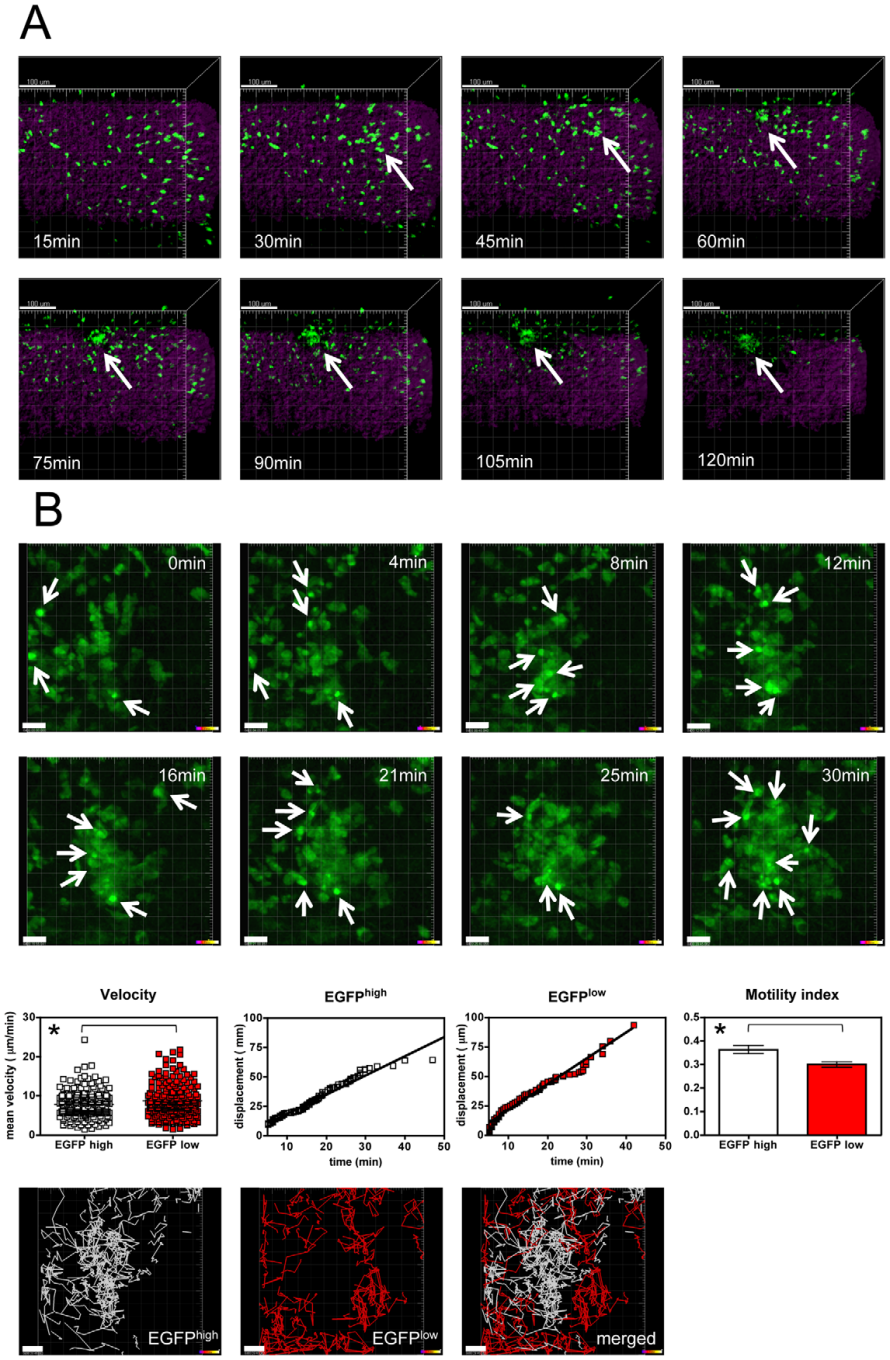
GMC cluster formation. A) Real time in vivo cell cluster formation (arrow) during a 2 h time interval. Individual pictures were taken at the indicated time points (Green channel: EGFP^+^ GMC cells, magenta channel: SHG of collagen structures). Scale bars represent 100 µm. Representative pictures are shown from movies that were made in three healthy control subjects and three subjects with CIA. B) Real time in vivo cell cluster formation during a 30 min time interval at higher magnification. Individual pictures of EGFP^+^ cells were taken at the indicated time points. Arrows point at individual cells with high EGFP fluorescence expression intensity. Scale bars represent 15 µm. Representative pictures are shown from movies that were made in three subjects with CIA. Quantification of cell velocity, displacement over time and the motility index (total displacement/cumulative path length) are shown for EGFP^high^ (defined as 30% of EGFP^+^ cells with highest mean fluorescence intensity of EGFP) and EGFP^low^ (defined as 30% of EGFP^+^ cells with lowest mean fluorescence intensity of EGFP) cells. Data points represent individual cells (EGFP^high^: n = 216; EGFP^low^: n = 202) compiled from three independent imaging session from individual joints of three CIA animals. Graph bars show mean±s.e.m. from the same experiments. Asterisks (*) indicate significant p values (p<0.05). Cumulative cell tracks are shown from one representative experiment for EGFP^high^, EGFP^low^ and both (merged) cell populations. Scale bars represent 15 µm.

When we analyzed the cell migration pattern over time we observed the formation of GMC clusters within the ROI of CIA but not of healthy animals (Figure 3A and Movie S3).

We detected the initiation of GMC cluster formation even within the short two hour time period of in vivo imaging.

Since we did not observe a breakup of cell clusters even after a prolonged time period of six hours, it appears that GMC cell clusters represent stable formations within the synovial tissue; indeed, given the reduced but still considerable migration activity of cells that do not agglomerate, these clusters may constitute an important component of the inflammatory tissue.

In lysM-EGFP mice endogenous neutrophils have been shown to be brightly EGFP labeled whereas monocytes and macrophages display a reduced EGFP fluorescence intensity [13], [14] (Figure S1). Accordingly, EGFP fluorescence intensity, to a certain extent, allows us to distinguish between neutrophils and monocytes and provides a first tool for the analysis of cell cluster composition. We found that analysis of GMC clusters at higher magnification over time revealed that both EGFP^high^ granulocytes and EGFP^low^ monocytes are involved in the formation of cell clusters (Figure 3B and Movie S4). We found that both EGFP^high^ and EGFP^low^ cells attracted to cell clusters were highly motile and interacted with each other throughout the observation period. To determine whether EGFP^high^ and EGFP^low^ cells also differ in their migratory behavior, we further performed comparative sub-analysis of EGFP^high^ and EGFP^low^ cells, defined as 30% of EGFP^+^ cells with highest mean fluorescence intensity of EGFP and 30% of EGFP^+^ cells with lowest mean fluorescence intensity of EGFP, respectively. In general, we observed a higher cell migration speed of GMC in regions of cell clusters (7.7±3.3 µm/min) as compared to the whole ROI (2.75±1.17 µm/min; p<0.0001). Within cell clusters we found that EGFP^high^ cells migrated at lower speed (7.8±3.3 µm/min, p = 0.029) and displayed an increased motility index (MI 0.36±0.26, p = 0.022) and a prolonged track duration (13±7 min, p = 0.013) as compared to EGFP^low^ cells (mean velocity: 8.7±4.1 µm/min; MI: 0.29±0.15; track duration: 10±5 min) (Figure 3B). Together these data suggest a higher motility of GMC inside cell clusters as compared to the overall ROI. Within cell clusters EGFP^high^ cells migrate slower and in a less random fashion and have a higher and prolonged confinement as compared to EGFP^low^ cells. In addition, analysis of cumulative cell tracks suggests a migration pattern that is characterized by a tendency of EGFP^high^ cells to reside in the center, whereas EGFP^low^ cells were frequently localized in the marginal zone of cell clusters (Figure 3B).

#### Analysis of cell cluster localization and composition

Despite the detailed information on cell migratory behavior, real time in vivo imaging can only provide some of the details of the organization and localization of cell clusters. Therefore, additional information on the composition and two-dimensional localization of these cells within the complex context of the anatomical environment of the inflamed joint can only be provided by other techniques. For this purpose, we expanded the analytic approach of real time in vivo imaging by subsequent ex vivo immunofluorescence, immunhistochemical and additional histomorphological analysis.

**Figure 4:**
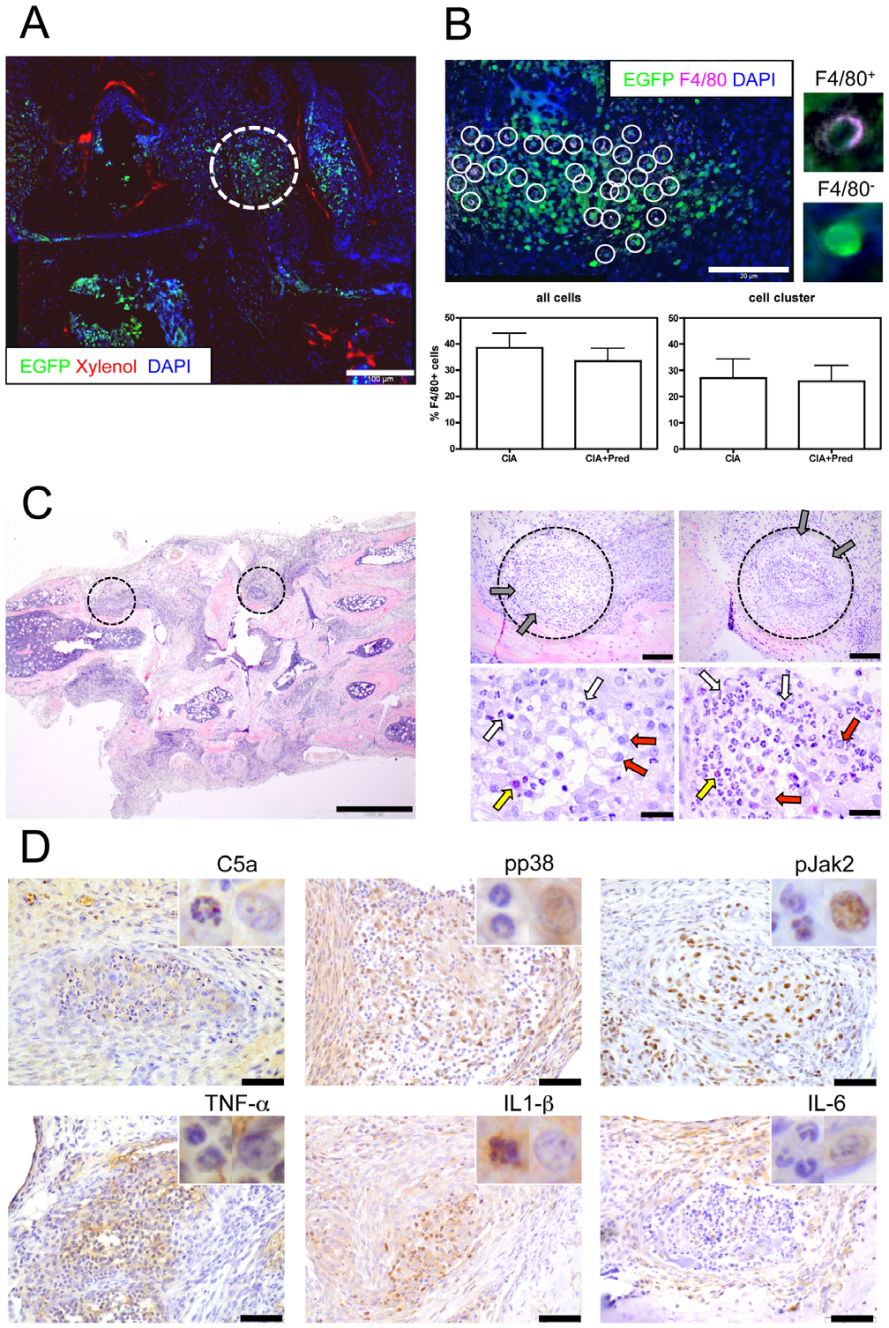
GMC cluster analysis. A) Immunofluorescence analysis of cryo sections stained with the bone tissue-specific dye Xylenol Orange and the nuclear dye DAPI. EGFP^+^ GMC cell cluster (green) are localized within the synovial tissue (white dotted circle, left picture). Scale bar represents 100 µm. Representative pictures are shown from experiments that were performed in three animals with CIA. B) Higher magnification of one GMC cell cluster (right picture). Cryo sections were stained with the monocyte marker F4/80 and DAPI. Circles indicate individual EGFP^+^F4/80^+^ monocytes. Inserts at higher magnification show representative examples of EGFP^+^F4/80^+^ and EGFP^+^F4/80^−^ cells. The majority of EGFP^+^ (green) cells within cell clusters are F4/80 negative neutrophil granulocytes. Scale bar represents 20 µm. Graph bars represent mean values±s.e.m. of F4/80^+^ monocytes of all EGFP^+^ cells analyzed, and of EGFP^+^ cells within cell cluster obtained from three individual experiments. No significant difference in the proportion of F4/80^+^ cells was observed between untreated (CIA) and prednisolone treated (CIA+Pred) CIA subjects. C) Representative HE staining of paraffin sections from three CIA subjects demonstrates cell cluster formation (circles). Cell clusters are surrounded by fibroblast like cells (grey arrows) and consist of neutrophil granulocytes (white arrows) and macrophages (red arrows). Occasionally eosinophil granulocytes (yellow arrows) can be detected. Scale bars represent 1000 µm (left picture), 100 µm (upper two right) and 2 µm (lower two right). D) Representative IH staining of paraffin sections from three CIA subjects for C5a, pp38 and pJak2 (upper row) and for the cytokines TNF-alpha, IL-1-beta and IL-6 (lower row). Inserts at higher magnification show representative examples of the respective staining on granulocytes (left) and monocytes (right). Scale bars represent 50 µm.

We sacrificed animals after in vivo imaging and prepared cryo sections of joints. We found that GMC clusters, which mainly consisted of EGFP^high^ cells, were predominantly detected within the inflamed synovial tissue but rarely in the immediate proximity or directly adjacent to bone tissues (Figure 4A).

In order to obtain further specific information about the composition of a cell cluster, we stained cryo sections of joints for the monocyte specific marker F4/80. We found that on average 38±17% of all EGFP^+^ GMC were F4/80^+^ monocytes, whereas the remainder were EGFP^+^ F4/80^−^ granulocytes (Figure 4B).

Histomorphological analysis of HE stained paraffin section further demonstrated that cell clusters were frequently detected within the synovial tissue of inflamed joints. Cell clusters were surrounded by a wall of fibroblast-like cells and consisted of neutrophils, monocytes/macrophages and very few eosinophilic granulocytes (Figure 4C); the finding that neutrophilic granulocytes were primarily located in the center of the aggregates and were surrounded by monocytes/macrophages confirms our observations from in vivo experiments and the analysis of cryo-sections where monocytes were seen at the marginal zone of the aggregates.

#### Analysis of the contribution of cell clusters to the inflammatory process

Importantly, we found that these clusters occurred in areas where complement staining (C5a) was prevalent (Figure 4D), suggesting that complement components (presumably related to immune complex formation) not only contribute to the immigration of GMC into the synovial tissue, but are also involved in capturing cells that provide a focus for cluster formation.

We stained paraffin sections for the presence of phosphorylated (p) p38 which represents a key mitogen-activated protein kinases (MAPK) associated with the induction as well as downstream effects of tumor necrosis factor alpha (TNF-alpha) and other proinflammatory cytokines [15]. We observed a positive staining for pp38 in monocytes/macrophages in cell clusters but not in granulocytes. We also performed stainings for pJak2, a member of the Janus family of kinases that are required for type I and II cytokine receptor signaling [16]. Again, within cell clusters, we detected a positive staining for pJak2 mainly in monocytes/macrophages and occasionally in granulocytes.

In line with these findings, within cell clusters, we detected a positive staining of monocytes/macrophages for TNF-alpha. In contrast we observed a positive staining of granulocytes and of the surrounding fibroblasts, but not of monocytes/macrophages, for IL-1-beta. Finally, we detected a positive staining for IL-6 in scattered monocytes/macrophages within cell clusters, in the surrounding fibroblasts but much more abundant in monocytes/macrophages of the adjacent tissue. Only occasionally did we observe positive staining for IL-6 in granulocytes within the cell clusters (Figure 4D).

#### Analysis of EGFP^low^F4/80^+^TRAP^+^ cells

**Figure 5:**
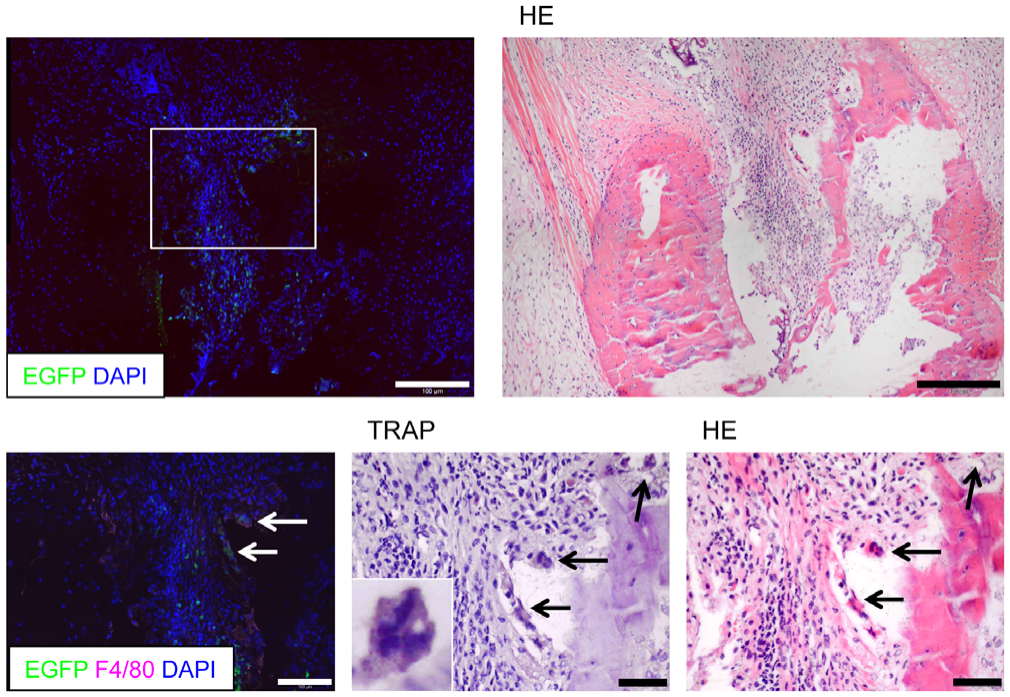
Combined sequential immunofluorescence/histomorphological analysis of monocytes/osteoclasts. Analysis of cryo sections stained with DAPI (left) and subsequent HE (right) staining (upper two pictures). A higher magnification of the insert demonstrates that individual EGFP^low^ cells (arrows) express F4/80 and TRAP, are multinucleated and localized in close vicinity to the bone tissue. A higher magnification insert in the middle pictures shows one EGFP^low^F4/80^+^TRAP^+^ cell. Representative pictures are shown from experiments that were performed in three animals with CIA. Scale bars represent 100 µm.

In addition to the detection of EGFP^low^F4/80^+^ monocytes in cell clusters, we observed multinucleated EGFP^low^F4/80^+^ cells in close contact to bone tissues. In order to further characterize this cell population, we destained cryo-sections of joints and restained with TRAP for the detection of osteoclasts. Thereafter, we destained the same sections again and restained them with hematoxylin and eosin (HE) for the assessment of synovial inflammation and bone erosions. Using this sequential staining approach we were able to identify EGFP^low^F4/80^+^ monocytes as TRAP^+^ osteoclasts localized in close vicinity of bone tissue and areas of bone erosion (Figure 5).

### Effects of prednisolone treatment on GMC migration

Interfering with the inflammatory and destructive arthritic response is of pivotal importance in rheumatoid arthritis. Glucocorticoids are a mainstay of therapy and while their mechanisms of action and inhibitory capacity regarding the activation of cytokines and other mediators of inflammation are well known from *in vitro* and *ex vivo* studies, whether these drugs also affect dynamic aspects of inflammatory cell behavior in the affected joint is not known. Therefore, we addressed the question whether therapeutic intervention with glucocorticoids translates rapidly into detectable changes in the migratory behavior of GMC or cellular composition of synovitis in animals with CIA. For this purpose we injected CIA animals with a single dose (0.25 mg) of prednisolone i.v. one hour after start of in vivo imaging. Afterwards we continued the imaging for two additional hours. We analyzed the same variables as before: cell migration (velocity, displacement over time, motility index and immigration) and compared them between untreated CIA subjects and CIA subjects with prednisolone treatment.

#### Cell speed

We determined the average speed of GMC movement through tissue during two hours of imaging immediately after prednisolone injection and compared it to that of untreated animals. We found that GMC in prednisolone-treated animals moved significantly slower (2.19±1.06 µm/min) as compared to cells in untreated CIA animals (2.75±1.17 µm/min; p<0.001)(Figure 2B).

#### Cell Immigration and Abundance

We observed comparable numbers of immigrating cells at initial time points in both untreated and prednisolone treated animals (Figure 2E). In line with an anticipated delayed effect of prednisolone treatment, we observed no significant difference for immigrating cell numbers at initial time points in the linear mixed-effects model (Table 1a). However, when we compared the slope of the two curves (i.e., the shared influence of group and time, determined by a model with interaction term), it became evident that prednisolone therapy significantly decreased the immigration of cells into the scanning area (Table 1b). Thus, our results indicate that prednisolone treatment exerted a time-delayed effect on cell migration that manifested itself in a significantly diminished absolute cell immigration over time in treated CIA subjects as compared to untreated CIA subjects.

#### Random versus directed cell movement

We did not detect differences in cell movement when we calculated the MI in prednisolone-treated CIA subjects as compared to CIA subjects (Figure 2C, 2D). We found that both, the average MI (CIA: 0.3±0.16%; CIA+Prednisolone: 0.3±0.16%) as well as the very low relative frequency of cells with an MI<0.8 (CIA: 0.83%, CIA+Prednisolone: 0.75%) were not significantly different from one another.

Together these data indicate that prednisolone treatment did not affect the directionality of GMC movement, but it did reduce GMC accumulation and overall GMC motility within the synovial tissue.

#### Effects of prednisolone treatment on GMC and cluster formation

When we analyzed cryo sections ex vivo we found that prednisolone treatment did not cause a significant difference in the overall percentage of monocytes (33±14% F4/80^+^ cells) among EGFP^+^ GMC as compared to untreated animals (38±17% F4/80^+^ cells) (Figure 4B). In addition, we found that cell clusters in prednisolone treated animals did not contain significantly different proportions of F4/80^+^ monocytes (26±15%) as compared to untreated CIA subjects (27±18%).

## Discussion

This study is among the first to apply real time in vivo imaging to the characterization of the cellular events involved in arthritis in general (reviewed in [17]) and GMC migration into the synovial tissue of inflamed joints in particular [6], [7]. Arthritis was induced by immunization with collagen type II in LysM-EGFP C57BL/6 mice in which granulocytes and monocytes constitutively express the fluorescent marker protein EGFP [11]. GMC displayed random movement within the synovial tissue and were found to form cell clusters that appeared to actively contribute to the inflammatory process in the synovial tissue. Subsequent in vitro analysis revealed the presence of osteoclast precursor cells at the synovial-bone junction and in areas of bone erosions. Prednisolone treatment reduced the rate of cell immigration into the synovium and reduced the overall GMC motility.

There are several issues involved in imaging the joint that require careful consideration in evaluating the utility of intravital imaging as a tool for research in this area. Firstly the joint, and even more so the inflamed joint, represents a multilayered anatomical structure composed of several different tissues with highly diverse characteristics. Among them are the hyperplastic synovial tissue itself, blood vessels, tendons and cartilage as well as bone tissue. In addition, the variable formation of lymphoid follicle like structures, lymphatic vessels and a so far barely analyzed network composed of fibroblastic reticular cells that, as in lymphoid structures, might substantially contribute to the guidance of immune cell migration [18] are also present. This extensive and spatially complex tissue environment contrasts with the limited volumes that can be imaged by multi-photon microscopy. One-photon (confocal) approaches can typically obtain data to a depth of only 80 µm from the exposed tissue surface; two-photon methods, as used in our experiments, while extending to depths of 200–300 µm or more in tissues [19], are still limited in their capacity to provide a comprehensive image in overall volume relative to the joint structure. It is, therefore, a difficult task to ensure that functionally identical regions of two different inflamed joints are imaged in successive experiments even in small animal models.

Secondly, the CIA model itself – as most murine disease models - displays certain inter-individual variability in regard to the extent of disease expression that needs to be taken into account.

Thirdly, labeling methods to highlight specific anatomical structures and micro-domains within tissues are currently still limited. The visualization of cells, collagen tissue of tendons and cartilage, blood vessels and newly formed bone tissue may not provide a precise idea of the exact anatomical context inside the joint during in vivo imaging, in particular under conditions of synovial inflammation.

To address this issue of sampling, sequential performance of in vivo imaging together with post-mortem analysis of previously scanned tissues to confirm in vivo data has been included in most imaging studies from the very beginning [20]. In contrast to lymphoid or other homogeneous tissues, however, the ex vivo analysis of joint tissues poses additional challenges. Usually rather lengthy fixation and decalcification of the bone tissue are required to make the bone flexible for the generation of paraffin sections. Unfortunately, this procedure can destroy fluorescent probes and, therefore, prevent the detection of EGFP^+^ GMC on paraffin sections. In consideration of these obstacles we decided that an approach that combines the analysis of extensive data volumes obtained from real time in vivo imaging with elaborate histomorphological and immunohistological post-mortem analysis on paraffin and/or cryo sections would be most suitable for the determination of GMC behavior in the complex anatomical setting of the inflamed joint. Our approach introduces new methodological aspects and has led to several novel insights.

Thus, we used a special tape transfer system that allows the generation of sections of snap-frozen, un-decalcified bone tissue. Moreover, we developed a sequential staining procedure that combines immunofluorescence, immunohistochemical, enzyme histochemical as well as conventional HE staining techniques on a single tissue section. This allows for the determination of the expression of multiple marker molecules on an individual cell as well as the analysis of the precise localization within the synovial tissue and/or anatomical context.

The exact assessment of the localization of immune cells in relation to anatomical structures, however, is of considerable importance, in particular for the development of arthritis. For example, localized inflammatory processes, characterized by the infiltration of the tendon sheaths by granulocytes and macrophages, frequently herald later spreading of inflammation and can be observed even before the onset of clinical signs of arthritis [21]. Likewise focal bone erosions in arthritic joints are mediated by osteoclasts [22], [23] and typically start at two principal sites: at the joint margins and in the subchondral bone [24]. The detection and analysis of immune cells together with the elaborate composition of the synovial tissue are, therefore, hard to achieve by in vivo imaging alone and require a combined approach.

In vivo imaging using the LysM-EGFP reporter strain produced results consistent with prior knowledge about the rare presence of GMC in the joint area of healthy rodents based on static imaging [4], [25]. However, it added new information on the dynamic behavior and showed that these rare cells migrated with an average speed of 3.11 µm/min. In contrast, substantially higher numbers of GMC were seen in the synovial tissue under inflammatory conditions; their motility and average speed of migration was lower (mean 2.75 µm/min) when compared to healthy subjects except for GMC in cell clusters where we observed the highest migratory velocities. Interestingly, comparable migration velocities have been reported for neutrophils in non-inflamed tissues [14], whereas slightly higher velocities of neutrophil movement have been observed in the footpad of mice under inflammatory conditions [26]. Differences in the type of inflammation (bacterial versus autoimmune), the type of imaged tissue (subcutaneous versus synovial) as well as the cell type being analyzed (isolated neutrophils versus both neutrophils and monocytes), however, might account for these minor differences.

The overall migratory behavior of GMC was found to follow random patterns except for the formation of cell clusters within the synovial tissue. This observation is reminiscent of other studies that described the aggregation of neutrophils and monocytes in cell clusters in lymph nodes [13], the skin [27] or in the interstitial tissue of the lung [14] under inflammatory conditions. In the latter of these studies, blood monocytes were found to mediate neutrophil extravasation and were necessary for cluster formation [14]. Likewise, in our study, cells clusters were found to consist of both granulocytes and monocytes with granulocytes apparently within the center and monocytes surrounding them. In addition, the migration of granulocytic cells at a reduced cell speed in the center of inflammatory cells cluster has been described by others as well [13], [14], indicating that the pattern of cell cluster formation within the synovial tissue may be similar to that seen in other inflammatory conditions. The detection of pp38 and pJak2 kinases in cell clusters together with the positive staining for TNF-alpha, IL-1-beta and IL-6 further suggests that the formation of cell clusters represents the most active component of the inflammatory process in the synovial tissue.

Interestingly, the combined immunofluorescence and histomorphological workup of articular cryo sections after in vivo imaging revealed that GMC clusters were located primarily within the synovial tissue but not next to bone tissues or more precisely in areas of explicit bone erosion. In contrast, we occasionally detected EGFP^low^ F4/80^+^ multinucleated cells with incipient TRAP expression directly adjacent to bone tissue and/or areas of bone erosions. In general mature osteoclasts are regarded to be TRAP^+^F4/80^−^ although osteoclasts can be derived from F4/80^+^ cells under in vitro conditions [28] and F4/80 expression has been shown on osteoclast precursor cells [29]. In addition, the transient generation of TRAP^+^F4/80^+^ osteoclast precursor cells has also been observed under in vitro conditions [30], [31]. One is, therefore, tempted to speculate that these cells represent immature monocyte-derived osteoclasts or at least osteoclast precursor cells at a transitional differentiation stage from monocytes to osteoclasts that are responsible for the active destruction of bone tissue.

In order to test whether our experimental setup is also qualified to detect and analyze the effects of therapeutic compounds we decided to treat arthritic animals with a single dose of prednisolone. The therapeutic use of glucocorticoids is well established in the treatment of several acute and chronic inflammatory diseases such as asthma, allergic rhinitis, rheumatoid arthritis and inflammatory bowel disease [32]. However, in contrast to the widespread use in the clinical setting and the multi-faceted spectrum of actions of these drugs, relatively little is known about the detailed mechanisms by which glucocorticoids exert their anti-inflammatory effects in the complex in vivo environment [33]. Most cellular responses to GC can be observed within two hours of exposure and some even within minutes [34], [35]. GC treatment, for example, was found to profoundly increase circulating neutrophil numbers and to enhance the survival and function of neutrophils [36]. Interestingly, we were also able to detect the effects of prednisolone treatment, in particular a lower GMC immigration and diminished cell velocity, already within a two hour observation period. On the other hand, effects on the composition of GMC aggregates or the extent of inflammation in general did not become evident within this time period and, therefore, need to be studied at later time points in future experiments.

In summary, our study introduces a variety of novel techniques for the analysis of cellular processes in inflamed synovial tissue. The comprehensive procedure of analyses, consisting of real time in vivo imaging using advanced light microscopy together with elaborate histomorphological ex vivo analysis, enabled us to characterize dynamic GMC migration in the anatomical context of inflamed joints at early disease stages. Furthermore, the sensitivity of this approach became apparent by the detection of therapeutic effects.

Therefore, the data presented herein can also effectively serve as a basis for future studies of immune cell biology as well as the assessment of new therapies in murine models of rheumatoid arthritis.

## Materials and Methods

### Animals

We obtained wild type (WT) female C57BL/6 mice from Charles River Laboratories (Charles River Inc., Boston, MA). We obtained C57BL/6 mice with the transgenic expression of enhanced green fluorescent protein (EGFP) in the murine lysoyzme M (lys) locus (lysM-EGFP) from Thomas Graf (Center for Genomic Regulation, CRG, Barcelona, Spain). We maintained all animals in our animal facility in accordance with institutional guidelines for animal welfare. We obtained approval for all animal experiments by a local ethical committee of the medical university of vienna (contract number: Zl. 3681/115-97/98). All surgery was performed under isoflurane anesthesia and all efforts were made to minimize suffering.

### Collagen Induced Arthritis (CIA)

We induced arthritis in 6–8 week old female WT C57BL/6 or lysM-EGFP C57BL/6 mice using an immunization procedure as described previously [37]. Briefly, we injected animals i.d. at the tail base with a total of 100 µl emulsion containing 100 µg chicken collagen type II (CII; Sigma Chemical Co., St. Louis, MO) and 250 µg M. tuberculosis (H37Ra; Difco Laboratories, Detroit, MI) in incomplete Freund's adjuvant (IFA; Sigma) and repeated this procedure on day 21.

### Clinical assessment

We assessed the mice for clinical signs of arthritis like paw swelling and grip strength as described previously [23], [38]. Briefly, we assessed paw swelling by using a semiquantitative score: 0 = no swelling, 1 = mild swelling of the toes and ankle, 2 = moderate swelling of the toes and ankle, and 3 = severe swelling of the toes and ankle.

We analyzed the grip strength of each paw on a wire of 3 mm diameter using a semiquantitative score: 3 = normal grip strength, 2 = mildly reduced grip strength, 1 = severely reduced grip strength, 0 = no grip at all. We found that on average 76±9% of animals developed signs of arthritis with a mean onset of 28±11 days after the first immunization. We used animals with paw swelling ≥2 for further experiments.

### Preparation and treatment of animals with CIA

We injected some animals s.c. with 2400 µg Xylenol-Orange (Sigma) for the visualization of newly formed bone tissue. For the visualization of blood vessels we injected Texas Red-conjugated bovine serum albumin (Texas Red-Alb; Invitrogen) i.v. immediately before imaging.

In selected animals we gave a single i.v. injection of 0.25 mg prednisolone-21-hydrogensuccinate-sodium (Merck) in phosphate buffered saline (PBS).

### Real time in vivo multi-photon microscopy

For in vivo microscopy we analyzed three animals in each group (healthy subjects, CIA subjects and prednisolone treated CIA subjects).

We anesthetized subjects with 1.5–2% Isoflurane mixed with 0.2 l O_2_ and 0.5 l air. We used a surgical microscope (Olympus, Hamburg, Germany) for the preparation of metatarsophalangeal joints.

We used an upright Leica TCS SP5 microscope (Leica Microsystems, Wetzlar, Germany) equipped with three confocal lasers (Argon, HeNe 543 and 633) and a Multi-Photon MaiTai Ti∶Sapphire laser (Spectra Physics, Newport Corp. Irvine, CA). For keeping the subject's body temperature at 35°C we installed a temperature box (‘The Cube and The Box’, Live Imaging Services, Basel, Switzerland) around the microscope's stage. We submerged the joint in medium (RPMI 1640 without phenol red; Sigma) for the use of a water immersion objective (HCX APO 20x/0.50 W U-V-I; Leica).

For fluorescence excitation we used the multi-photon MaiTai laser (Spectra Physics) tuned at 800 nm for the detection of EGFP^+^ GMC, Xylenol Orange and Texas Red-Albumin. We used Second Harmonic Generation for the visualization of collagen fibers in tendons and cartilage as described previously [39]. We collected images in 4D-movies with typical voxel size = 1.5×1.5×15 µm and a volume dimension = 775×775×250–300 µm depending on the visible signal. For 4-dimensional (4D) data sets we captured 3-dimensional stacks in healthy and untreated animals with CIA and prednisolone-treated CIA subjects for 2 h with 5 min intervals. For higher magnification of cell clusters we scanned the images with a zoom factor 3 for 30 min with 30 sec intervals.

### Data analysis and statistics

We transferred raw image datasets to Imaris software (Bitplane) for automatic 3D object tracking to retrieve cell spatial coordinates over time and data of cell kinetics was exported to Graph Pad Prism 4.0 (Graph Pad, La Jolla, CA) and SPSS software (IBM Corp., Somers, NY) for further analysis. We performed simple comparative analyses between healthy subjects, CIA subjects, and prednisolone treated CIA subjects using Student's t-test for data that passed normality test (alpha = 0.05). All data are shown throughout the text as mean values±s.d. unless indicated otherwise. To compare between the three groups over time we used a linear mixed-effects model including the random factor animal and the interaction between group and time.. We analyzed the models in the free online software R (http://www.r-project.org/) and regarded p values<0.05 to be significant. For dynamic display of imaging data sets, we composed image sequences in Imaris to produce video clips. We used the Surpass option of Imaris to display individual cells as spheres and the SHG signal as surfaces wherever indicated.

### Histological assessment of arthritis

#### Histomorphology

We assessed the joint histology as described previously [23]. We fixed hind paws in 4.0% formalin for six hours and then decalcified them in 14% EDTA (Sigma) for seven days at 4°C. We stained serial paraffin sections (2 µm) of hind paws with hematoxylin and eosin (HE) for assessment of synovial inflammation and bone erosions, with toluidine blue (TB) for cartilage degradation and with a leukocyte acid phosphatase staining kit (Sigma) for tartrate-resistant acid phosphatase (TRAP) to detect osteoclasts. We analyzed the sections with the use of an Axioskop 2 microscope (Zeiss, Marburg, Germany) equipped with a digital camera and an image analysis system (Osteomeasure; Osteometrics, Decatur, GA) which allows absolute quantification of areas in histologic sections. The sum of the areas of inflammation for each single mouse was calculated by evaluating all tarsal joints. The same H&E-stained sections were analyzed similarly for the quantification of erosions. In addition, given the established role of osteoclasts in the pathogenesis of local bone erosions, the number of osteoclasts was counted in TRAP-stained serial sections.

#### Immunohistochemistry

We performed an enzymatic digestion of paraffin sections with Proteinase K (Roche) for 5 minutes at 37°C. We blocked endogenous peroxidase with 0.3% hydrogen peroxide in PBS for 10 min. We stained sections with antibodies against Ly6B.2 (clone 7/4; Serotec, Oxford, UK), C5a (Abbiotec, San Diego, CA), pp38 (clone P38-TY, Sigma), pJak2 (clone E132, Epitomics, Burlingame, CA), TNF-alpha (Abbiotec), IL-1-beta (R&D Systems, Minneapolis, MN) and IL-6 (Abbiotec). Afterwards we incubated the sections for 30 min with biotinylated rabbit anti–rat IgG (Vector, Burlingame, CA), and detected the reaction by incubating with the VECTASTAIN-Elite reagent (Vector). We developed the color using diamino- benzidine (Sigma).

#### Immunofluorescence

For immunofluorescence analysis we fixed undecalcified hind paws overnight in 4.5% paraformaldehyde and prepared cryo sections using the CryoJane Tape-Transfer System (Instrumedics Inc. Richmond, IL). For the analysis of monocytes, we stained sections with anti-F4/80 (clone Cl:A3-1) antibody (Serotec) and with goat-anti-rat Alexa 647 (Invitrogen, Carlsbad, CA) as second step antibody. We performed nuclear staining using DAPI (Invitrogen). We mounted the slides with Prolong Gold anti-fade (Invitrogen).

For the sequential analysis of individual joint sections, we removed the cover slips after immunofluorescence data acquisition, and stained sections with TRAP. We performed microscopy analyses as described above. Afterwards, we removed the cover slips again and destained sections with 1% HCl-Ethanol. Subsequently, we stained sections with HE and performed microscopy analyses.

#### Image acquisition and processing

We took pictures with an Axioskop 2 microscope (Zeiss) equipped with a digital camera (Olympus). We processed images with Cell∧F (Olympus) and Adobe Photoshop 8.0.1 (Adobe, San Jose, CA).

## Supporting Information

Figure S1 **EGFP expression in Granulocytes and Monocytes.** Peripheral blood mononuclear cells from LysM-EGFP animals were analyzed for the expression intensity of EGFP. A) Higher EGFP fluorescence expression intensity can be observed in granulocytes compared to monocytes as determined by flow cytometry (FACS) and fluorescence microscopy (B). C) EGPF^high^ cells are found throughout the entire z-stack. One representative z-stack is shown from experiments that were performed in three animals with CIA. Arrows indicate EGFP^high^ cells along the z-axis. Bars represent 50 µm. The graph shows a balanced distribution of EGFP^high^ cells along the z-axis.
(TIF)

Movie S1 **GMC cell movement in healthy C57BL/6 lysM-EGFP animals.** Green channel: EGFP^+^ GMC cells, magenta channel: SHG of collagen structures. The Surpass option of Imaris was used to display individual cells as spheres and the SHG signal as surfaces. Z-stacks were collected every 5 minutes. The total time of data collection represented in the movie is one hour.
(MPG)

Movie S2 **GMC cell movement in C57BL/6 lysM-EGFP animals with CIA.** Green channel: EGFP^+^ GMC cells, magenta channel: SHG of collagen structures. The Surpass option of Imaris was used to display individual cells as spheres and the SHG signal as surfaces. Z-stacks were collected every 5 minutes. The total time of data collection represented in the movie is one hour.
(MPG)

Movie S3 **GMC cell cluster formation in C57BL/6 lysM-EGFP animals with CIA.** Green channel: EGFP^+^ GMC cells, magenta channel: SHG of collagen structures. The Surpass option of Imaris was used to display the SHG signal as surfaces. Z-stacks were collected every 5 minutes. The total time of data collection represented in the movie is two hours.
(MPG)

Movie S4 **High magnification of GMC cell cluster formation in C57BL/6 lysM-EGFP animals with CIA.** Green channel: EGFP^+^ GMC cells. Z-stacks were collected every 30 seconds. The total time of data collection represented in the movie is 30 minutes.
(MPG)
