# Supplementary figures and images for: A Dynamic Real Time In Vivo and Static Ex Vivo Analysis of Granulomonocytic Cell Migration in the Collagen-Induced Arthritis Model
Source: PLoS One. 2012 Apr 18;7(4):e35194. doi: 10.1371/journal.pone.0035194 (PMC3329447; doi:10.1371/journal.pone.0035194)

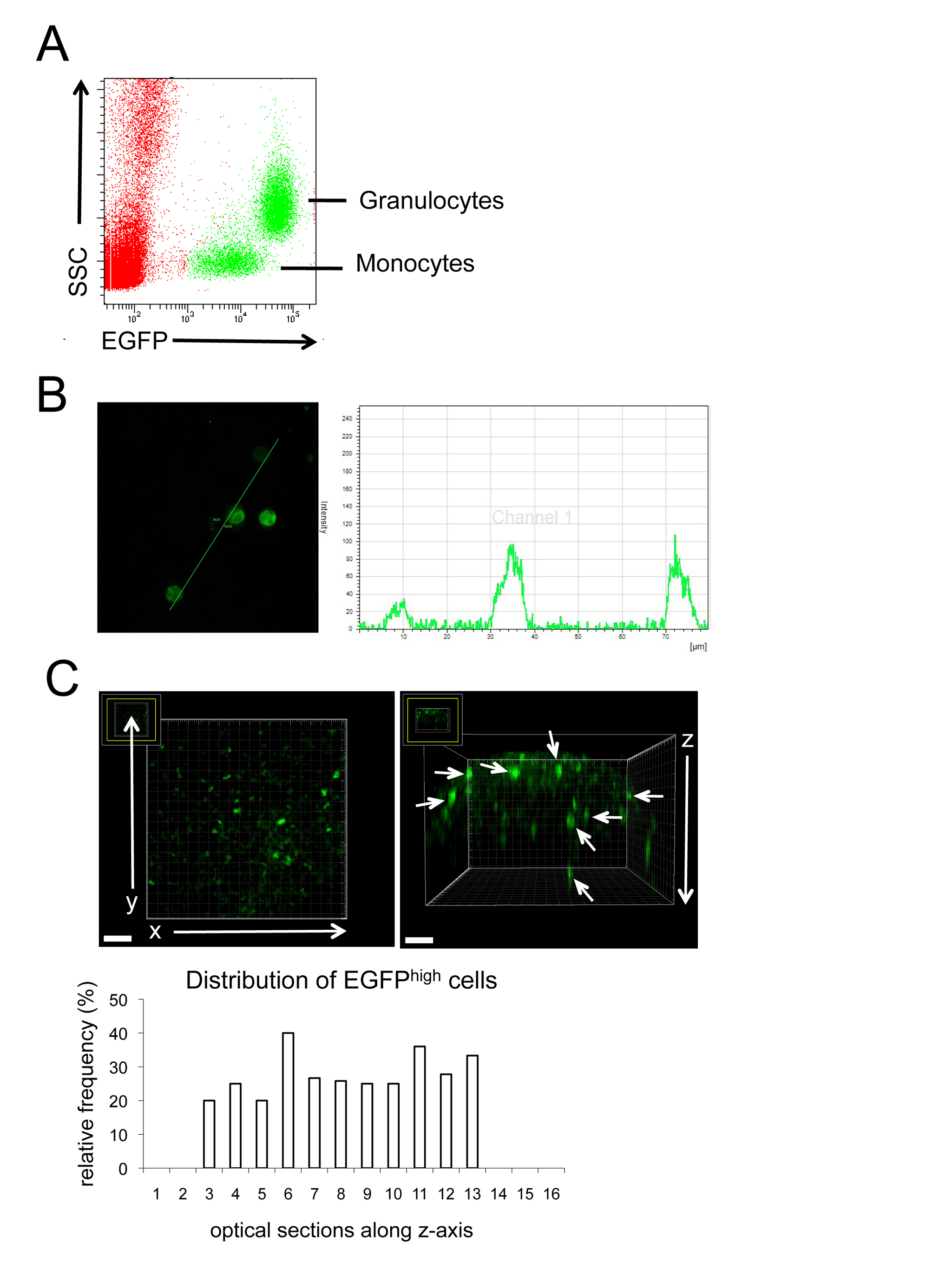

Supplement: Figure S1 — EGFP expression in Granulocytes and Monocytes. Peripheral blood mononuclear cells from LysM-EGFP animals were analyzed for the expression intensity of EGFP. A) Higher EGFP fluorescence expression intensity can be observed in granulocytes compared to monocytes as determined by flow cytometry (FACS) and fluorescence microscopy (B). C) EGPFhigh cells are found throughout the entire z-stack. One representative z-stack is shown from experiments that were performed in three animals with CIA. Arrows indicate EGFPhigh cells along the z-axis. Bars represent 50 µm. The graph shows a balanced distribution of EGFPhigh cells along the z-axis. (TIF) [file pone.0035194.s001.tif]
